# VHA-19 Is Essential in *Caenorhabditis elegans* Oocytes for Embryogenesis and Is Involved in Trafficking in Oocytes

**DOI:** 10.1371/journal.pone.0040317

**Published:** 2012-07-02

**Authors:** Alison J. Knight, Nicholas M. Johnson, Carolyn A. Behm

**Affiliations:** Research School of Biology, College of Medicine, Biology and Environment, Australian National University, Canberra, Australian Capital Territory, Australia; Universidade de São Paulo, Brazil

## Abstract

There is an urgent need to develop new drugs against parasitic nematodes, which are a significant burden on human health and agriculture. Information about the function of essential nematode-specific genes provides insight to key nematode-specific processes that could be targeted with drugs. We have characterized the function of a novel, nematode-specific *Caenorhabditis elegans* protein, VHA-19, and show that VHA-19 is essential in the germline and, specifically, the oocytes, for the completion of embryogenesis. VHA-19 is also involved in trafficking the oocyte receptor RME-2 to the oocyte plasma membrane and is essential for osmoregulation in the embryo, probably because VHA-19 is required for proper eggshell formation via exocytosis of cortical granules or other essential components of the eggshell. VHA-19 may also have a role in cytokinesis, either directly or as an indirect effect of its role in osmoregulation. Critically, VHA-19 is expressed in the excretory cell in both larvae and adults, suggesting that it may have a role in osmoregulation in *C. elegans* more generally, probably in trafficking or secretion pathways. This is the first time a role for VHA-19 has been described.

## Introduction

Parasitic nematodes are a significant burden on human health [1] and cause large losses to agriculture [2]. Nematode-specific genes are of interest because they may provide selective drug targets for treatment of parasitic nematodes, or highlight essential pathways in these important organisms. The free-living nematode *Caenorhabditis elegans* is an excellent model in which to study nematode-specific genes because its genome is fully sequenced and well annotated (unlike many of the parasitic nematodes of interest), and it is easy to manipulate experimentally. We identified VHA-19 in a screen for essential nematode-specific proteins in *C. elegans*. VHA-19 satisfied the criteria of the screen because it had less than 30% sequence identity over 150 amino acids to any mammalian proteins; it had 30% or greater sequence identity to predicted proteins from nematode parasites; and had a reported maternal sterile and lethal phenotype when gene expression was silenced by RNA interference (RNAi) [3], [4], [5].

This work shows that VHA-19 has a role in *C. elegans* reproduction. The reproductive system of the *C. elegans* hermaphrodite consists of two U-shaped gonad arms that each terminate with a spermatheca, connecting to a central uterus. Oocytes and spermatozoa are generated from the primordial germ cell, which resides at the distal end of each gonad arm and proliferates by mitosis. The proliferation of the primordial germ cell is controlled by the distal tip cell (Kimble & White, 1981). Hermaphrodite *C. elegans* produce sperm during the fourth larval (L4) stage, and switch to producing oocytes in early adulthood. Oocytes migrate from the distal gonad and line up in the proximal gonad where they are squeezed one by one through the spermatheca. Once fertilized in the spermatheca, the embryo is passed into the uterus and subsequently laid via the vulva. The egg hatches into the first larval stage, and the worm progresses through four larval stages in total before moulting into the adult hermaphrodite. Male *C. elegans* also occur; they exclusively produce sperm and are capable of mating with hermaphrodite *C. elegans*. When this occurs, male sperm are used preferentially [6], [7].

In addition to reproduction, we also demonstrate that VHA-19 is involved in osmoregulation and osmotic support of the embryo. The embryo is considered protected from osmotic shock by the inner lipid layer of the eggshell [8], which is thought to be formed by the extrusion of cytoplasmic granules [9]. Osmotic support is important for early cytokinesis in the embryo, particularly in hypertonic or hypotonic media [10]. A lack of osmotic protection can lead to cytokinesis defects [10].

Here we show that VHA-19 is essential in *C. elegans* oocytes for the production of viable progeny and in early larval development. Within the oocyte, VHA-19 is involved in the trafficking of the oocyte receptor protein RME-2 to the plasma membrane. Since some *vha-19(RNAi)* embryos appear to be fertilized but are osmotically sensitive, we also hypothesize that VHA-19 is required for integrity of the eggshell and, possibly also cytokinesis, in the early embryo.

## Results

### Key Features of VHA-19

VHA-19 is a 451 amino acid protein (www.wormbase.org) for which the bioinformatics applications TMPred [11] and HMMTOP [12] predict a transmembrane domain between amino acids 408–426. Also, the ProP 1.0 server [13] predicts that there is an N-terminal signal cleavage site between amino acids 16 and 17 in VHA-19 and that it is cleaved by furin between amino acids 159 and 160. Interestingly, the furin cleavage site and signal sequence are also present in translated expressed sequence tags of putative VHA-19 homologues from parasitic nematodes (not shown).

Based on a small region of similar sequence to the mammalian protein Ac45, VHA-19 is predicted to bind to the V0 subunit of the vacuolar ATPase (www.wormbase.org). This is because mammalian Ac45 has been shown to bind to several subunits of the V0 domain [14]. However, any association between VHA-19 and the *C. elegans* V-ATPase is yet to be shown experimentally.

### *vha-19* Expression is Essential for *C. elegans* Post Embryonic Larval Development and Embryogenesis

To investigate the role of VHA-19 in *C. elegans*, we fed worms on *vha-19* dsRNA, commencing at the first larval stage (L1) and also from the fourth larval stage (L4). We used RNA interference (RNAi) because *vha-19(tm2205)* mutant *C. elegans* are inviable (not shown).

*C. elegans* fed *vha-19* dsRNA from the L1 stage arrested and died as larvae by the L3/L4 stage (Table 1), indicating that VHA-19 is essential for early larval development. Feeding with *vha-19* dsRNA, commencing at the L4 stage, resulted in adults that appeared grossly normal. However, only 26% (n = 233) of the progeny produced by these adults hatched into larvae (Table 2), and all of these F1 larvae arrested and died by the L3 stage.

**Table 1:** *C. elegans* arrest as larvae when fed on *vha-19* dsRNA from the first larval stage.

dsRNA	24 h L2/L3/L4 (%)	Total no. worms	48 h L2/L3/L4 (%)	Total no. worms	72 h L2/L3/L4/adult (%)	Total no. worms	larval arrest (%)
control	99/1/0	753	0/6/94	656	0/0/0/100	790	0
*vha-19*	98/2/0	747	0/99/1	738	0/99/1/0	655	100

**Figure 1:**
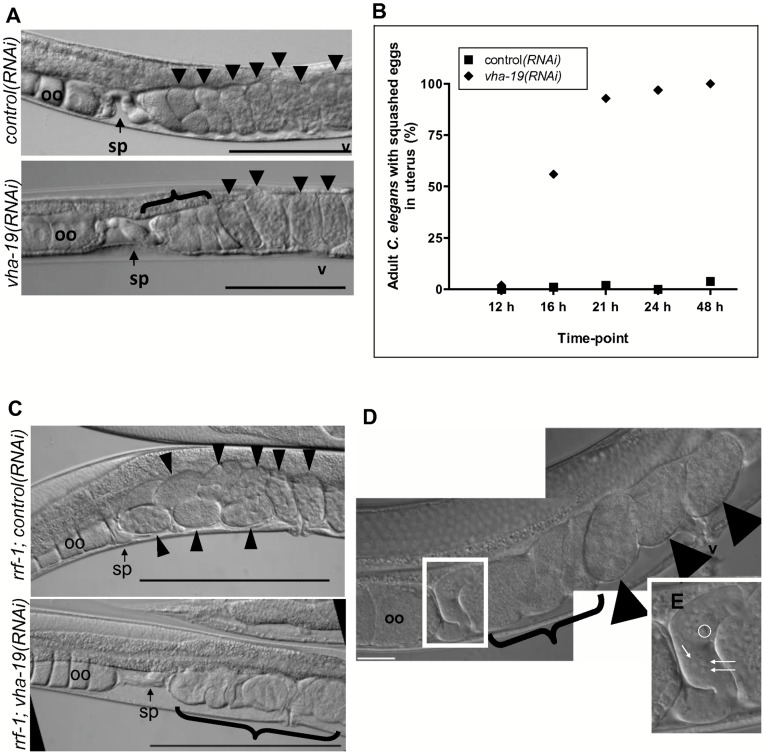
*vha-19(RNAi)* adults lay compressed eggs despite the presence of sperm in the spermatheca. Wild type (A-B, D) or *rrf-1* (C) *C. elegans* were fed control or *vha-19* dsRNA from the L4 stage for 16–48 hours. A) The proximal gonad of a *control(RNAi)* and *vha-19(RNAi)* adult after feeding on dsRNA for 16 hours. B) The percentage of *control(RNAi)* or *vha-19(RNAi)* adults that had one or more compressed (squashed) eggs present in their uterus from 16–48 h. At least 89 worms were scored for each experimental group at each time point. C) The proximal gonads of *rrf-1(pk1471) C. elegans* after feeding on dsRNA for 24 hours. D) A typical proximal gonad of a wild type *C. elegans* that was fed *vha-19* dsRNA from the L4 stage for 24 hours. Note the presence of newly released, compressed eggs in the uterus (bracket), even though there are sperm in the spermatheca (enclosed by a white box and magnified in E. Examples of sperm are indicated by arrows or enclosed by a white circle). In all images black arrowheads indicate normal embryos, and a bracket indicates compressed eggs. The oocytes (oo) and spermatheca (sp) are also indicated. v =  vulva. Scale bars represent 100 µm in A and C and 10 µm in D.

To investigate why the eggs of *vha-19(RNAi)* adults failed to hatch we examined the reproductive organs of these worms and observed compressed eggs present in their uterus (Figure 1A). These compressed eggs were first observed after 16 hours of feeding on *vha-19* dsRNA and were typically located closest to the spermatheca (Figure 1A–B). Eggs with a normal appearance were also observed in the uterus of *vha-19(RNAi)* adults, but they were usually located close to the vulva, indicating that they had emerged from the spermatheca earlier than the compressed eggs (Figure 1A).

**Table 2:** *vha-19(RNAi)* adult *C. elegans* lay eggs that seldom hatch.

	Embryonic arrest in eggs laid in:		
dsRNA	0–24 hour time period	24–48 hour time period	48–72 hour time period
control	0% (n = 386, P0 = 9)	0% (n = 1347, P0 = 12)	0% (n = 437, P0 = 8)
*vha-19*	74% (n = 233, P0 = 10)	100% (n = 736, P0 = 12)	100% (n = 95, P0 = 7)

n =  total number of progeny scored.

P0 =  number of individual broods that were scored.

The compressed eggs observed in the uteri of *vha-19(RNAi)* adults probably corresponded to embryos that failed early embryogenesis. In agreement with this hypothesis, no *vha-19(RNAi)* embryo hatched into larvae if it was laid beyond 24 hours of exposure to dsRNA (Table 2), and beyond this time point nearly all *vha-19(RNAi)* adults had switched to producing compressed eggs (Figure 1B). Thus, in addition to post-embryonic larval development, expression of *vha-19* is also required for early embryogenesis and/or earlier events in *C. elegans* reproduction.

### VHA-19 is Required in the Germline for Embryogenesis

To determine whether *vha-19* expression was required exclusively in the germline for the production of normal embryos, we silenced *vha-19* expression in *rrf-1* mutant *C. elegans* [NL2098]. RRF-1 is essential for RNAi in somatic tissue, but is not required for RNAi in the germline [15]. Similar to wild type *C. elegans* adults fed *vha-19* dsRNA (Figure 1A), 94% (n = 83) of *rrf-1*; *vha-19(RNAi)* adults had compressed eggs present in their uterus after feeding on dsRNA for 24 hours from L4 (Figure 1C) whereas the compressed-egg phenotype was almost absent (1%; n = 76) in *rrf-1; control(RNAi)* adults (Figure 1C). This implies that VHA-19 has an essential role in the germline, because silencing expression of *vha-19* in the oocytes and/or sperm alone was sufficient to induce the compressed-egg phenotype.

### A Sperm Defect in *vha-19(RNAi)* Adults is Unlikely to Explain the Presence of Compressed Eggs

Given that *vha-19* expression is required in the germline, we examined the spermatozoa, oocytes and process of ovulation in more detail in *vha-19(RNAi)* adults to identify abnormalities. Spermatozoa observed in the spermatheca of *vha-19(RNAi)* adults appeared to be normal, were present at the same time as compressed eggs (Figure 1D) and were still present after 72 hours of exposure to dsRNA. We therefore examined the ability of these spermatozoa to trigger ovulation, which occurs via the secretion by sperm of major sperm protein [16], [17].

**Figure 2:**
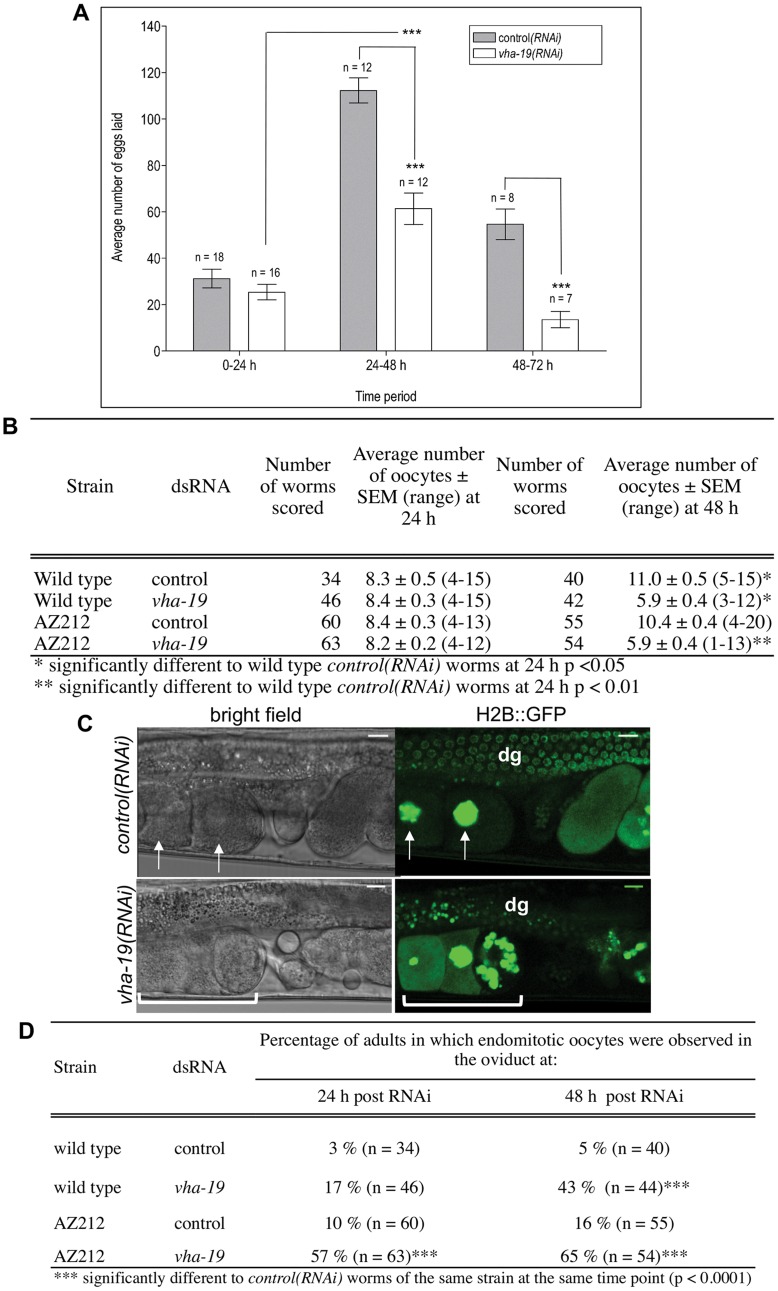
*vha-19(RNAi)* adults produce fewer oocytes beyond 24 hours of exposure to *vha-19* dsRNA. A) The average number of eggs laid every 24 hours by *control(RNAi)* or *vha-19(RNAi)* adults feeding on dsRNA. Error bars represent the standard error of the mean (s.e.m). ***indicates p<0.001. B) The average number of oocytes in the proximal oviduct of either wild type or AZ212 (pie-1::H2B::GFP) *C. elegans* fed on control or *vha-19* dsRNA for 24 or 48 hours. C) The proximal oviduct of AZ212 *C. elegans* fed control or *vha-19* dsRNA for 24 hours. White arrows indicate normal oocyte nuclei. Endomitotic oocytes in the oviduct are indicated by a square bracket. Note that both oocytes with multiple nuclei (most proximal oocyte in square bracket), and oocytes in which the chromosomes have started to decondense prematurely (indicated by the green background of the two most distal oocytes in the square bracket), are endomitotic. The irregular, bright, particulate dots in the distal gonad (dg) are autofluorescent gut granules. Scale bars indicate 10 µm. D) The percentage of endomitotic oocytes observed in the oviducts of wild type or AZ212 *C. elegans* fed control or *vha-19* dsRNA from the L4 stage for 24 or 48 hours.

The rate of ovulation in *vha-19(RNAi)* adults was investigated by counting the number of eggs laid every 24 hours. As oocytes must pass though the spermatheca to be laid (whether fertilized or not), comparing the average number of eggs laid between individual *control(RNAi)* and *vha-19(RNAi)* worms allowed the rate of ovulation to be determined, and thus, whether there was a backup in the oviduct of *vha-19(RNAi)* worms. Interestingly, the average number of eggs laid by *vha-19(RNAi)* adults was not significantly different from *control(RNAi)* adults in the first 24 hours of exposure to dsRNA (p>0.05; Figure 2A), but was significantly lower than the number of eggs laid by *control(RNAi)* worms in the subsequent time periods (p<0.001; Figure 2A). This indicates that the rate of ovulation was not reduced in the time period where compressed eggs were first observed in the uterus of *vha-19(RNAi)* worms (0–24 hours; Figure 1B), but was reduced in subsequent time periods. There are three possible explanations for this: first, while the sperm that were observed in the spermathecae of *vha-19(RNAi)* worms at the 24 hour time-point (Figure 1D) were probably able to trigger ovulation normally, *vha-19(RNAi)* oocytes may have been released at a slower rate beyond this time point. Second, there may have been fewer sperm in the spermatheca of *vha-19(RNAi)* adults overall, which would result in the rate of ovulation decreasing more quickly in *vha-19(RNAi)* adults. Third, *vha-19(RNAi)* oocytes may have become less responsive to sperm in the later time periods.

### VHA-19 has a Role in the Oocytes

Notably, *vha-19(RNAi)* adults had significantly fewer oocytes in the oviduct than *control(RNAi)* worms at the 48-hour time point (p<0.05; Figure 2B), despite the presence of spermatozoa in the spermatheca. This was the case for both wild type *C. elegans* and H2B::GFP *C. elegans* [AZ212] fed *vha-19* dsRNA (Figure 2B) (H2B::GFP *C. elegans* were used to better visualize the nuclei of individual oocytes). This suggests that the rate of oocyte production declines prematurely in *vha-19(RNAi)* adults and explains why fewer eggs were laid by *vha-19(RNAi*) adults compared with *control(RNAi)* adults in the 48–72 hour time period (Figure 2A).

Not only did the rate of oocyte production decline in *vha-19(RNAi)* adults compared with *control(RNAi)* adults; the oocytes also exhibited uncontrolled replication (endomitosis) (Figure 2C). As early as the 24-hour time point, when the rate of oocyte production did not differ between the experimental groups (Figure 2A), a significantly greater number of *vha-19(RNAi)* adults had endomitotic oocytes in the oviduct compared with *control(RNAi)* adults (p<0.0001; Figure 2D). Endomitotic oocytes were visualized as oocytes with multiple nuclei, or oocytes in which the chromosomes had begun to decondense, indicating that nuclear envelope breakdown had occurred [18] (Figure 2C). Endomitotic oocytes were also present in the oviducts of *vha-19(RNAi)* adults at the 48-hour time point, at a higher frequency (Figure 2D). These observations suggest that some *vha-19(RNAi)* oocytes are defective before fertilization.

Collectively, the requirement of *vha-19* expression in the germline for normal embryogenesis (Figure 1C), the decline in oocyte production (Figure 2A–B), and the presence of endomitotic oocytes in the oviducts of *vha-19(RNAi)* adults (Figure 2C–D), suggest that VHA-19 has an essential role in the development of the oocytes. To confirm this, a mating experiment was designed. Virgin female *fog-2 C. elegans* [CB4108], which do not produce sperm and, thus, are unable to reproduce without mating [19], were fed on *vha-19* or control dsRNA and mated with male *fog-2 C. elegans* fed control or *vha-19* dsRNA. The resulting progeny from each mating were then tracked throughout their development and compared.

Only 13% (n = 415) of the progeny sired by *fog-2*; *control(RNAi)* males reached adulthood when they were produced by *fog-2*; *vha-19(RNAi)* females (Table 3), whereas all progeny sired by *fog-2; control(RNAi)* males and produced by *control(RNAi)* females reached adulthood (Table 3). Hence *fog-2*; *control(RNAi)* males were capable of siring normal progeny. Similarly, the number of eggs laid by *fog-2*; *vha-19(RNAi)* females mated with *fog-2*; *control(RNAi)* males was significantly lower than the average number of eggs laid by *fog-2*; *control(RNAi)* females mated with *fog-2; control(RNAi)* males (p<0.01; Table 3).These results suggest that both the proportion of progeny that reached adulthood and the number of eggs that were laid were independent of the dsRNA on which the male was fed, but rather depended on the dsRNA on which the female was fed. Thus, VHA-19 was required in the oocytes rather than the sperm, indicating that expression of *vha-19* in the oocytes is essential for successful embryogenesis.

**Table 3:** The progeny produced by mating *fog-2* male *C. elegans* with *fog-2* female *C. elegans* fed on either control or *vha-19* dsRNA.

				Phenotypes of progeny		
dsRNA fedto male	dsRNA fed to female	Number of broods scored	Average brood size ± SEM (range)	Total number of progeny scored	Embryonic lethality (%)	Progeny that developed into adults (%)
control	control	18	77±8 (5–115)	1428	0	100
control	*vha-19*	15	28±5 (5–62)∧*	415	87	13∧*
*vha-19*	*vha-19*	16	21±5 (5–65)∧	321	85	15∧

∧Significantly different to average brood size (p<0.01) or number of progeny that reached adulthood (p<0.0001) for *fog-2(q71) V*; *control(RNAi)*l x *fog-2(q71) V*; *control(RNAi)* mating.

* Not significantly different to average brood size/number of progeny that reached adulthood for *fog-2(q71) V*; *vha-19(RNAi)* x *fog-2(q71) V*; *vha-19(RNAi)* mating (p>0.05).

### *vha-19(RNAi)* Oocytes do not Endocytose Vitellogenin

To explore further the cause of defective oocytes in *vha-19(RNAi)* adults we assayed the ability of *vha-19(RNAi)* oocytes to endocytose vitellogenin, using a transgenic strain in which one of the *C. elegans* vitellogenins, VIT-2, is fused to GFP [DH1033] [20]. Vitellogenins are lipid-binding proteins, synthesized in the intestine, that are secreted as yolk granules from the intestine into the pseudocoelom (body cavity), where they diffuse into the gonad via pores in the somatic gonadal sheath cells [21], [22]. In the somatic gonadal sheath cells, yolk granules come into direct contact with developing oocytes, which endocytose the granules using the low density lipoprotein receptor, RME-2, by receptor-mediated endocytosis [20], [21].

**Figure 3:**
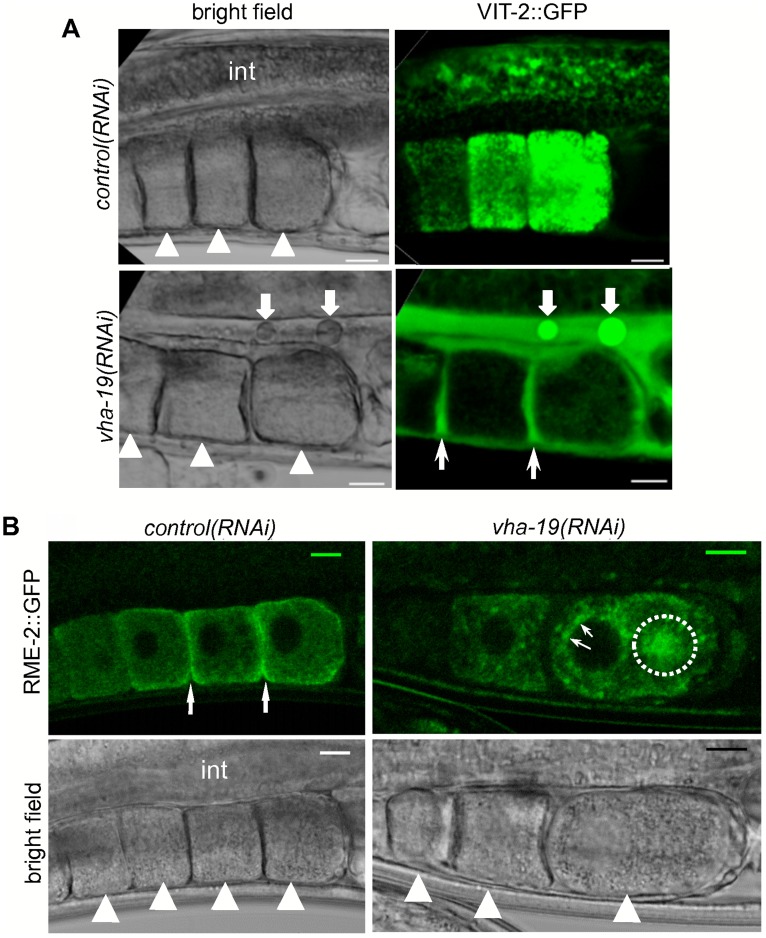
VHA-19 is required for uptake of vitellogenin via trafficking of the RME-2 receptor. A) VIT-2::GFP *C. elegans* [DH1033] were fed control or *vha-19* dsRNA from the L4 stage for 48 hours and the oocytes examined. VIT-2::GFP did not reach the oocytes in adults fed *vha-19* dsRNA, instead it accumulated between the oocytes (thin arrows). Fat arrows indicate agglomerates of VIT-2::GFP. B) RME-2::GFP was not trafficked to the plasma membrane in RME-2::GFP; *vha-19(RNAi)* adults (arrows and dotted circle), whereas RME-2::GFP did reach the plasma membrane in worms fed control dsRNA (thin arrows between oocytes). Worms were fed *vha-19* or control dsRNA for 48 hours. Arrowheads in bright field panels indicate individual oocytes. Scale bars represent 10 µm.

VIT-2::GFP did not reach its final destination, the germline, in 82% (n = 117) of VIT-2::GFP; *vha-19(RNAi)* adults. Instead, VIT-2::GFP was predominantly found in the pseudocoelom of these worms, between individual oocytes (Figure 3A). Thus, VIT-2::GFP reached the surface of *vha-19(RNAi)* oocytes but was not endocytosed. As this phenotype was only observed in 7% of VIT-2::GFP; *control(RNAi)* adults (n = 89), these results suggest that VHA-19 might be involved in either receptor-mediated endocytosis directly, or in the trafficking of RME-2, the receptor required for this process.

### VHA-19 is Required for Trafficking of RME-2 to the Oocyte Plasma Membrane

To determine whether VHA-19 is involved in the traffic of RME-2, the localization of RME-2::GFP in the oocytes was compared between transgenic *C. elegans* adults [RT408] fed control or *vha-19* dsRNA from the L4 stage. Examination of the oocytes of worms from each experimental group showed that the majority of RME-2::GFP aggregated around the nucleus of *vha-19(RNAi)* oocytes and did not reach the plasma membrane (Figure 3B). The aggregates that formed at the periphery of the nucleus (Figure 3B) resembled previously reported “Golgi –like” blocks in secretion [23], suggesting that a block in the trafficking of RME-2::GFP (which occurs via a secretory pathway) [20] may have occurred. In contrast, RME-2::GFP was localized to the plasma membrane of the oocytes in RME-2::GFP; *control(RNAi) C. elegans* (Figure 3B). The observed block in the trafficking of RME-2 could explain why *vha-19(RNAi)* adults are unable to endocytose sufficient vitellogenin (Figure 3A).

### *vha-19(RNAi)* Embryos have a Faulty Eggshell

We questioned whether VHA-19 had a role in trafficking or secretion at other stages of reproduction and embryonic development. Because *vha-19(RNAi)* embryos were compressed (Figure 1A), we investigated their eggshell. The *C. elegans* eggshell forms shortly after fertilization [7], provides mechanical and osmotic support for the embryo [24], and has been shown to be faulty when expression of known components of the secretory pathway are silenced by RNAi [20].

**Figure 4:**
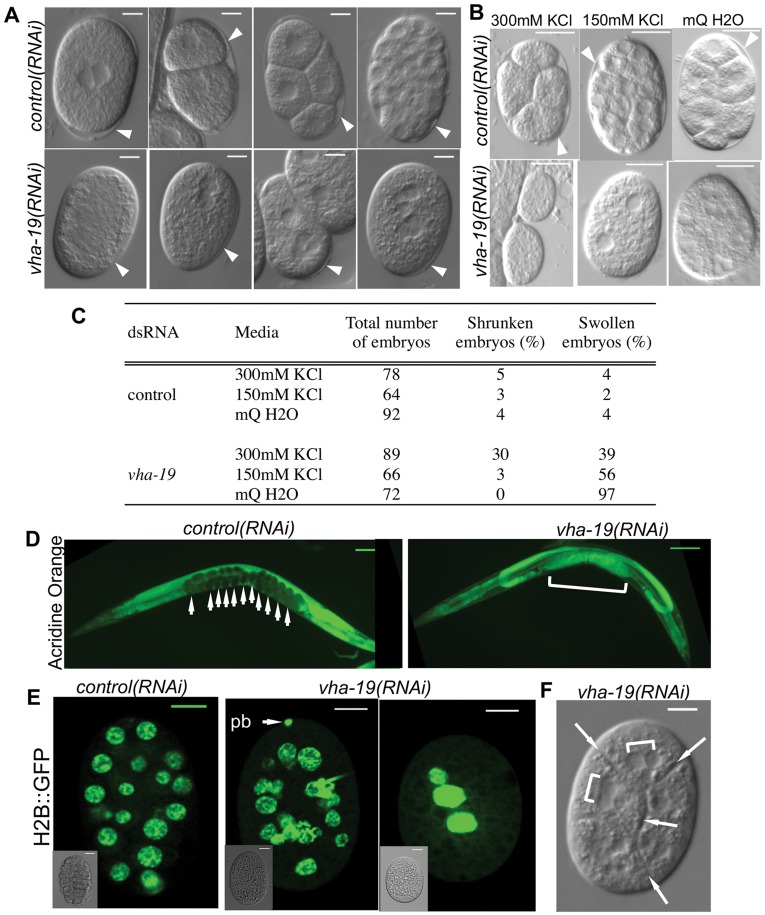
*vha-19(RNAi)*embryos are osmotically sensitive and appear to have a cytokinesis defect. A) Embryos cut out of the uterus of wild type (A-B,F) or pie-1::H2B::GFP [AZ212] (E) adult *C. elegans* fed control or *vha-19* dsRNA for 24 hours. Note the presence of an eggshell in all embryos in A (arrow heads), despite the random clusters of nuclei and compression of some *vha-19(RNAi)* embryos. Note also the presence of a polar body (pb) in a *vha-19(RNAi)* embryo in E, even though the nuclei are not cellularized (inset, bright field image), and that in F, non-cellularized, small clusters of nuclei (square bracket) are surrounded by cleavage furrows (arrows) in the *vha-19(RNAi)* embryo. C) The percentage of *control(RNAi)* and *vha-19(RNAi)* embryos that swelled or shrunk in deionized water (mQ H2O), 150 mM potassium chloride (KCl) and 300 mM KCl. Examples of embryos from this osmolarity experiment are shown in B. D) Embryos in the uterus of adults fed control or *vha-19* dsRNA for 48 hours and stained with Acridine Orange. Note that embryos in the uterus of *control(RNAi)* adult excluded Acridine Orange (arrows) whereas the embryos in the uterus of the *vha-19(RNAi)* adult were permeable to Acridine Orange (bracket). Scale bars represent 100 µm in D and 10 µm in A-B,E and F.

Regardless of the time point at which they were isolated, approximately 80% (n = 59) of isolated *vha-19(RNAi)* embryos had a visible eggshell (Figure 4A). Some *vha-19(RNAi)* embryos had a clearly visible eggshell even when they were compressed (Figure 4A), indicating that the structural fidelity, but not the presence, of the eggshell is dependent on *vha-19* expression. In agreement with this hypothesis, *vha-19(RNAi)* embryos were osmotically sensitive in hypertonic and hypotonic media (Figure 4B), from which protection would normally be provided by the eggshell, and had a tendency to swell within their eggshell in all media types (Figure 4B–C), suggesting that osmoregulation was also abnormal in these embryos.

The osmotic sensitivity of *vha-19(RNAi)* embryos was demonstrated by a significantly higher proportion of *vha-19(RNAi)* embryos shrinking when exposed to 300 mM potassium chloride (KCl) (hypertonic) than *control(RNAi)* embryos in 300 mM KCl (p<0.0001) or *vha-19(RNAi)* embryos in 150 mM KCl (isotonic) (p<0.0001) (Figure 4C). Similarly, a significantly higher proportion of *vha-19(RNAi)* embryos swelled in deionised water (hypotonic) than *control(RNAi)* embryos in deionised water (p<0.0001) or *vha-19(RNAi)* embryos in 150 mM KCl (p<0.0001) (Figure 4C). In addition, we found that in all media types, a significant proportion (at least 39%) of *vha-19(RNAi)* embryos swelled to fill the eggshell (p<0.0001; Figure 4B–C), which suggests that *vha-19(RNAi)* embryos have a defect in osmoregulation more generally. This observation is in agreement with results from a genome-wide screen in which *vha-19(RNAi)* embryos were scored as having a general osmolarity defect which was further described as “embryo fills eggshell” [25].

*vha-19* was recently identified as a candidate gene in a screen for permeable embryos [26]. Thus *vha-19(RNAi)* embryos might be osmotically sensitive because their eggshells were permeable. Rappeleye and colleagues [27] have shown that permeability of the embryo can reflect a defect in the lipid layer of the eggshell. While 11% (n = 136) and 34% (n = 71) of *vha-19(RNAi)* embryos were permeable to Trypan Blue after 24 hours and 48 hours of exposure to dsRNA, respectively, the majority of isolated *vha-19(RNAi)* embryos were impermeable. Hence the lipid layer may be intact. However, many *vha-19(RNAi)* embryos laid after 48 hours of exposure to dsRNA disintegrated in the medium when they were isolated, which may have represented a population of *vha-19(RNAi)* embryos that were permeable. We therefore determined whether *vha-19(RNAi)* embryos in the uterus were permeable to the dye Acridine Orange at this late time point. These *in utero* embryos were permeable (Figure 4D), suggesting that VHA-19 may be involved in the secretion of the lipid layer of the eggshell in the embryo. However, as most *vha-19(RNAi)* embryos laid at earlier time points were impermeable, it is possible that the contents of the uterus of *vha-19(RNAi)* adults after longer periods of exposure to dsRNA represented unfertilized eggs, which would have lacked the permeability barrier. It is also possible that mechanical pressure in the uterus rendered *vha-19(RNAi)* embryos permeable.

### *vha-19(RNAi)* Embryos Exhibit Signs of Defective Cytokinesis

An intact eggshell is important for early cytokinesis events in the embryo [27], [28], [29]. In particular, successful cytokinesis in wild type embryos requires an impermeable eggshell to provide protection from osmotic pressure: embryos with permeable eggshells exhibit cytokinesis defects in hypotonic and hypertonic media, but not in isotonic media [10]. Because *vha-19(RNAi)* embryos had a defect in osmoregulation in addition to being osmotically sensitive (Figure 4C), we hypothesized that they may exhibit cytokinesis defects as well. We found that eggs cut out of the uteri of *vha-19(RNAi)* adults had multiple nuclei that varied in number from two upwards (Figure 4A–B,E–F). Interestingly, some *vha-19(RNAi)* embryos resembled the dead embryos produced when expression of a known subunit of the *C. elegans* V-ATPase, *vha-8*, was silenced [30]. In 11% (n = 91) of isolated *vha-19(RNAi)* embryos, furrows were present that randomly separated clusters of nuclei (Figure 4F), which were never observed in *control(RNAi)* embryos. These furrows resembled cytokinesis defects observed when wild type embryos with permeable eggshells were placed in hypotonic media [10], as well as cytokinesis defects described in *rab-11(RNAi)* embryos [31] and *syn-4(RNAi)* embryos [32], which are genes involved in the secretory pathway and membrane fusion, respectively. Interestingly, a polar body was clearly visible for a small proportion of *vha-19(RNAi)* embryos (Figure 4E), indicating that they were fertilized and that cytokinesis of the oocyte pronuclei had successfully occurred.

### Tissue Expression Pattern of VHA-19

**Figure 5:**
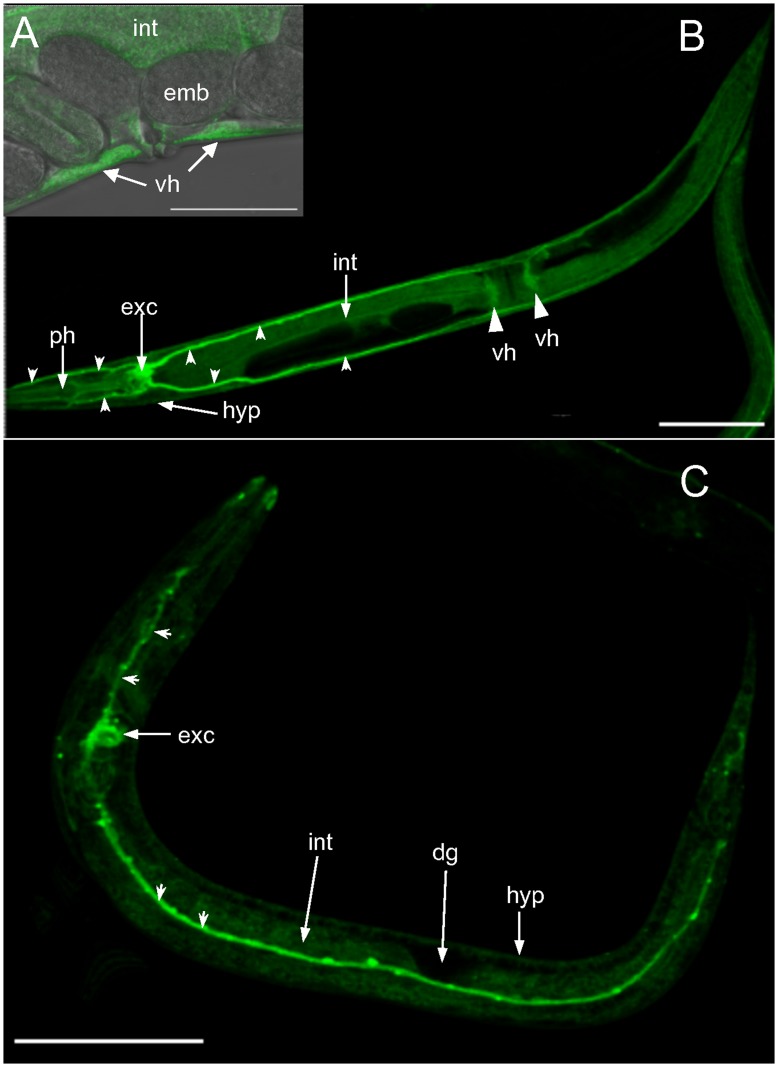
Expression of GFP directed by the presumed *vha-19* promoter in transgenic vha-19p::GFP *C. elegans*. A) High magnification of the vulva region of adult vha-19p::GFP *C. elegans* [WT029] showing that *vha-19* is expressed in vulval hypodermis (vh) and the intestine (int) but apparently not in early embryos (emb). The image is an overlay of a bright field and fluorescence image. B) An adult vha-19p::GFP *C. elegans* showing that *vha-19* is expressed in the pharynx (ph), hypodermis (hyp), intestine (int), vulval hypodermis (vh) and excretory cell, consisting of the excretory cell body (exc) and the long, thin parallel canals either side of the cell body (indicated by small arrow heads). C) Fluorescence image of a vha-19p::GFP larva showing that *vha-19* is expressed in the hypodermis (hyp) and intestine (int) but is apparently absent from the developing gonad (dg). *vha-19* is also expressed in the excretory cell body (exc) and canals (thin arrowheads) of vha-19p::GFP larvae. The orientation of the larva is such that only one excretory canal is visible. Scale bar in A represents 50 µm and 100 µm in B and C.

Since *vha-19(RNAi)* embryos were osmotically fragile and exhibited osmoregulation defects, we determined whether *vha-19* mRNA is expressed in tissues that control osmoregulation. We constructed a transgenic strain [WT029] in which a fragment of the presumed *vha-19* promoter sequence was fused to a GFP reporter and detected GFP in the excretory cell, intestine and hypodermis of vha-19p::GFP larvae and adults (Figure 5). These are tissues in which other known osmoregulation genes are expressed [33]. We could not detect *vha-19* expression in the germline (Figure 5), but transgenes are often silenced in the *C. elegans* germline [34], [35]. Indeed, there is consistent evidence from genome-wide expression studies that *vha-19* is both maternally and embryonically expressed [36], [37], [38] and in two recent studies *vha-19* mRNA has been detected in the germline [39] and in germline precursor cells [40]. To confirm the GFP expression pattern we attempted to create an antibody against VHA-19, but binding of this antibody was non-specific (not shown).

## Discussion

We have characterized the physiological and developmental roles of the novel *C. elegans* protein VHA-19 and have shown that it is essential in the oocytes for successful embryogenesis. This role somewhat resembles the described role of known V-ATPase subunits in *C. elegans* reproduction [30], [41], which is consistent with the hypothesised association between VHA-19 and the *C. elegans* V-ATPase. As well as the oocytes, VHA-19 is likely to have an important role in the developing embryo. This is because most *vha-19(RNAi)* embryos were clearly fertilized, as indicated by the presence of an eggshell (Figure 4A), which forms after fertilisation, and, in some embryos, a polar body (Figure 4E). Despite being fertilized, these embryos failed to complete embryogenesis. We therefore hypothesize that in this case a post-fertilization defect occurred during formation of the eggshell and/or cytokinesis, because *vha-19(RNAi)* embryos were osmotically fragile and responded abnormally to osmotic media (Figure 4B–C), and because some *vha-19(RNAi)* embryos resembled those observed by others when a cytokinesis defect occurs (Figure 4E–F) [32].

There are likely to be two explanations for the compressed eggs observed in the uterus of *vha-19(RNAi)* adults (Figure 1A). Eggs laid by *vha-19(RNAi)* adults after 24 hours of exposure to dsRNA are likely to be compressed because the structure of their eggshell is compromised, as indicated by their osmotic sensitivity (Figure 4B–C) and their compressed nature (Figure 4A). Eggs laid by *vha-19(RNAi)* adults after longer periods of exposure to dsRNA (and observed in the uterus) are likely to be compressed because the oocytes were defective, and so may not have been fertilized. In support of this explanation, eggs observed in the uterus of *vha-19(RNAi)* adults after 48 hours of exposure to dsRNA had often mixed together and could not be distinguished individually (e.g. Figures 1C, 4D).

An explanation for the osmosensitivity of, and general osmoregulation defect observed in, *vha-19(RNAi)* embryos could be that VHA-19 is involved in the trafficking of cortical granules. These form in developing oocytes and are exocytosed in anaphase I of oocyte meiosis, shortly after oocytes are activated by major sperm protein [42]. Disrupted trafficking of cortical granules results in embryos with defective eggshells and osmolarity defects [42], as observed in *vha-19(RNAi)* embryos (Figure 4B–C). Further, as VHA-19 was required for proper trafficking of the receptor RME-2 in oocytes (Figure 3B), and RME-2 is trafficked via the secretory pathway, a role for VHA-19 in the traffic or exocytosis of cortical granules is possible.

If VHA-19 were involved in a secretory pathway this could be relevant to cytokinesis as well, as regression of the cleavage furrow can occur when secretion from the Golgi to the plasma membrane is inhibited in embryos [31] and when hemicentin (a protein that is secreted into the pseudocoelom from the intestine) is absent from the germline [43]. Interestingly, when embryos fail to complete cytokinesis in the early stages of embryogenesis, nuclear division continues, resulting in embryos with multiple nuclei that are not cellularized [29], [44], which was observed for *vha-19(RNAi)* embryos (Figure 4A–B,E–F). Thus, is it possible that VHA-19 may be important for cytokinesis via a role in the secretory/trafficking pathway. Alternatively, since cytokinesis can be disrupted by osmotic pressure [10], and *vha-19(RNAi)* embryos are osmotically fragile, this may explain why some *vha-19(RNAi)* embryos appear to have a defect in cytokinesis.

We attempted to film *vha-19(RNAi)* embryos to ascertain precisely when in cytokinesis defects might occur, but could not detect actively dividing nuclei. In a few rare instances we observed pronuclei in *vha-19(RNAi)* embryos fusing and attempting to divide, but the cleavage furrow regressed and the nuclei collapsed together, which supports the hypothesis that VHA-19 may be involved in a critical secretion step in cytokinesis. Since most *vha-19(RNAi)* embryos had multiple nuclei (Figs 4A–B,E–F), this implies that nuclear division was not prevented. While it has been shown that providing osmotic support for embryos can rescue their cytokinesis defects [10], we could not rescue cell division using 150 mM KCl (a medium used for osmotic support of fragile embryos [28]) or Egg Buffer [45]. An intact eggshell is required for extrusion of the polar body and partitioning of P granules [10], [28], but we observed polar bodies in at least a few *vha-19(RNAi)* embryos (Figure 4E). Thus, while *vha-19(RNAi)* embryos are osmotically sensitive and clearly have defects in osmoregulation (Figure 4B–C), it is not clear whether the failure of cytokinesis in these embryos is an indirect result of osmotic sensitivity or because VHA-19 has a separate role in cytokinesis.

It is also possible that VHA-19 may have a role in cytokinesis in the oocytes, because endomitotic oocytes were observed in the oviducts of *vha-19(RNAi)* adults (Figure 2C–D), and *vha-19(RNAi)* adults produced fewer oocytes overall (Figure 2B). Interestingly, these phenotypes were also observed when expression of known subunits of the V-ATPase were silenced [41], although the onset of the phenotypes was earlier and more severe when individual V-ATPase genes were silenced. Efficient germline proliferation requires functioning cytokinesis [46], and expression of *vha-19* mRNA has been detected in the germline precursor cell [40], suggesting that VHA-19 may have a role in germline proliferation. Loss of function of cytokinesis genes also induces multinucleate oocytes [46], which were observed in the oviducts of 20% of *vha-19(RNAi)* adults (not shown), but this was not significantly different from *control(RNAi)* adults (10/46 vs. 2/34). If there was a defect in cytokinesis in *vha-19(RNAi)* oocytes, this could also be an indirect consequence of oocyte osmotic fragility.

In summary, we propose that VHA-19 is involved in trafficking of molecules in the oocytes and the embryo that are essential for osmotic homeostasis, and possibly, cytokinesis. Such a role for VHA-19 would explain why multinucleate, but non-cellularized, embryos that were osmotically sensitive were observed earlier in the egg-laying cycle and also why endomitotic oocytes and a late decline in oocyte production were observed. This is the first time that a role for VHA-19 has been described in *C. elegans*.

## Materials and Methods

### *C. elegans* Strains and Culture

*C. elegans* were cultured using standard methods [47] at 20°C unless stated otherwise. The N2(Bristol) strain was used as the *C. elegans* wild type strain. Transgenic and mutant strains were obtained from the Caenorhabditis Genetics Center (University of Minnesota, Minneapolis, MN, USA) and were as follows: NL2098: (*rrf-1(pk1417)*); AZ212: (*unc-119*(ed3) ruIs32[unc-119(+) pie-1::GFP::H2B] III); CB4108: (*fog-2(q71)V*); RT408: (*unc-119*(ed3) III; pwIs116[unc-119(+) + rme-2::GFP]); *unc-119*(ed3); and DH1033: (bls1[vit-2::GFP + rol-6(su1006)]).

### RNAi Experiments

*C. elegans* were fed on HT115(DE3) *E. coli* transformed with an RNAi feeding vector as previously described [3], [48]. Synchronized L1 or L4 *C. elegans* were placed on RNAi plates expressing either negative control or *vha-19* dsRNA and incubated at 20°C for 12–72 hours. The negative control was pCB19, which expresses dsRNA from a fragment of the *Arabidopsis thaliana* light-harvesting gene, lhcb4.3, that has no significant sequence identity to any gene in the *C. elegans* genome [49]. *C. elegans* were fed on *vha-19* dsRNA using a construct (IV-1D10) purchased from Source BioScience (Nottingham, UK).

### Microscopy of *C. elegans*

Whole *C. elegans* and eggs were visualized using a Leica DMLB microscope fitted with Nomarski optics (Leica Microsystems GmbH, Wetzlar, Germany) and a halogen lamp for fluorescent microscopy, or a Leica ultraviolet SP2 Confocal microscope (Leica Microsystems GmbH, Wetzlar, Germany). Images were captured using a SPOT RT-SE6 1.4 MP Slider digital camera and SPOT Advance software (Meyer Instruments, Houston, Texas, USA) for the Leica DMLB microscope and Leica confocal software (Leica Microsystems GmbH) for the SP2 UV confocal microscope. Images were analysed using either ImageJ software or the GIMP 2.2 software.

### Time Course of the Number of Eggs Laid and Fate of their Progeny

Individual L4 *C. elegans* were fed on control or *vha-19* dsRNA, incubated at 20°C and transferred to fresh RNAi plates every 24 hours for a total of 72 hours. The number of eggs and progeny produced by each adult in each time period were counted and monitored for 96 hours beyond hatching, with the developmental stage of progeny scored every 24 hours.

Embryonic arrest was defined as eggs that had not hatched after 96 hours. The average number of eggs laid at each time point was compared among experimental groups using a one-way analysis of variance (ANOVA) and a Tukey-Kramer multiple comparisons test using InStat (GraphPad Software, La Jolla, California, USA). Results were pooled from three separate experiments performed at least in duplicate.

### Counting Oocytes in the Oviduct and Scoring for Endomitotic Oocytes

Wild type or AZ212 *C. elegans* were fed on control or *vha-19* dsRNA from the L4 stage for 24 and 48 hours. The oviducts of the resulting individual adult worms were examined using either Nomarski microscopy (wild type) or confocal microscopy (AZ212) for endomitotic oocytes or multinucleate oocytes, and the number of oocytes in the proximal oviduct was also counted. Endomitotic oocytes were defined as oocytes with multiple nuclei, or oocytes in which the chromosomes had started to decondense, indicating that a breakdown of the nuclear envelope had occurred. Numbers of oocytes were compared using a Dunn’s multiple comparisons test (InStat). The incidence of endomitotic oocytes was compared among experimental groups using a Fisher’s exact test (nonparametric ANOVA: InStat). Results were pooled from three separate experiments.

### Mating of Males with Females

Individual virgin female and male *fog-2 C. elegans* [CB4108] were fed on control or *vha-19* dsRNA from the L4 stage for 24 hours at 20°C. The following crosses were then set up on individual plates seeded with OP50 *E. coli*: 1) *fog-2* male and female, both fed on control dsRNA, 2) *fog-2* male fed on control dsRNA, *fog-2* female fed on *vha-19* dsRNA and 3) *fog-2* male and female both fed on *vha-19* dsRNA. To ensure that mating occurred, three males were used in each cross with one female. Virgin *fog-2* females were also picked to individual OP50 plates to ensure that these females could not produce eggs without mating; no eggs were ever observed (not shown). All mating plates were incubated at 20°C for 24 hours, adults were removed, and the number of eggs laid by each female (including any progeny that had already hatched into larvae) was determined. The progeny resulting from each mating were then followed for a further 8–9 days, with their stage of development (L1–adult) scored every 24 hours. The experiment was performed three times with at least five replicates of each cross per experiment and results were pooled for each experimental group. The percentage of worms that reached adulthood was compared among each group using a Fisher’s exact test (Instat). The average number of eggs laid in each experimental group was compared using a Kruskal-Wallis Test (nonparametric ANOVA, Instat).

### Eggshell Assays

Wild type or AZ212 *C. elegans* L4s were fed either control (pCB19) or *vha-19* (IV-1D10) dsRNA and incubated for either 24 or 48 hours at 20°C. Approximately ten *vha-19(RNAi)* or *control(RNAi)* adults were picked into separate depression slides containing 10 µl of 150 mM KCl (Sigma-Aldrich, St. Louis, MO, USA) as recommended by Johnston et al. [28] and the sharp edge of a 26 G needle (BD, Franklin Lakes, New Jersey, USA) was used to cut adult worms in half, allowing the eggs to spill out into the medium. Eggs were scored for the presence of an eggshell (defined as a clear, translucent ring around the egg). The experiment was repeated twice for each time-point and results from experiments were pooled for each time-point. The presence of an eggshell was also determined using 10 µl Egg Salts buffer [45].

### Acridine Orange Staining of Eggs in the Uterus

*C. elegans* were fed on *vha-19* or control dsRNA from the L4 stage for 48 hours, harvested by flooding plates with phosphate buffered saline (PBS), centrifuged and stained with 200 µl of 100 µg/ml Acridine Orange (Sigma-Aldrich) in 1% (v/v) dimethyl sulfoxide (Sigma-Aldrich) and incubated in the dark with agitation for 4–5 hours. Worms were washed with PBS four times to remove excess dye. Eggs in the uteri of adult *C. elegans* were visualised at excitation wavelength 490 nm and detected at 530 nm. The experiment was repeated and results were pooled.

### Osmotic Sensitivity of Embryos

Wild type *C. elegans* were fed control or *vha-19* dsRNA, commencing at the L4 stage, for 24 hours. At least six adults were cut at the head with a 26 G needle (BD) to release the embryos into each of 10 µl of deionised water, 150 mM KCl (Sigma-Aldrich) and 300 mM KCl (Sigma-Aldrich) on an agarose pad. Embryos were then visualised using a Leica DMLB microscope with Nomarski optics and scored for shrinking or swelling. The incidence of eggs that swelled or shrunk in the media was compared among experimental groups using a Fisher’s exact test (InStat). Results were pooled from two separate experiments.

### Generating Transgenic Strains

The transgenic vha-19prom::GFP construct was cloned using Gateway® cloning system [50]. Primers were designed to amplify a region encompassing 761 bp upstream of the *vha-19* ATG start codon and the first 30 bp of sequence of the *vha-19* exon 1. Primers were flanked with the attB4 and attB1R recombination sites (Forward primer: 5′CATGGTTAAGAGCGTGCTGGCGTCAC; Reverse primer: 5′ATGAGCGAGAAAACGGCAAAAAGTACCCTCAT). The 791 fragment was amplified from isolated *C. elegans* genomic DNA using KOD polymerase (Merck Chemicals, Darmstadt, Germany) in a standard reaction. The resulting polymerase chain reaction product was cloned into pDONR(P4-P1R) in a BP reaction (Invitrogen Australia; Mulgrave, Victoria, Australia) and then into pDESTDD04 using a LR reaction (Invitrogen Australia), using half the total reaction volumes recommended by the manufacturer. The final product was confirmed by sequencing and transformed into *C. elegans* using microparticle bombardment of *unc-119*(ed3) *C. elegans.* The resulting genotype of strain WT029 was *unc-119*(ed3), wtEx029[y55H10a.1::gfp, unc-119(+)].
